# Awareness and knowledge of HPV, cervical cancer, and vaccines in young women after first delivery in São Paulo, Brazil - a cross-sectional study

**DOI:** 10.1186/1472-6874-10-35

**Published:** 2010-12-22

**Authors:** Cristina H Rama, Luisa L Villa, Sonia Pagliusi, Maria A Andreoli, Maria C Costa, Aline L Aoki, Adhemar Longatto-Filho, José Eluf-Neto

**Affiliations:** 1 Hospital Maternidade Leonor Mendes de Barros, Sao Paulo, Brazil Av. Celso Garcia, 2477; 03015-000; Belenzinho; 2 Virology Department of Ludwig Institute for Cancer Research, Sao Paulo, Brazil; 3 Formerly with Immunizations, Vaccines and Biologicals Department, World Health Organization, Geneva, Switzerland; 4 Faculdade de Medicina da Universidade de Santo Amaro, Sao Paulo, Brazil; 5 Laboratório de Investigação Médica (LIM) 14, Departamento de Patologia, Faculdade de Medicina, Universidade de São Paulo, Sao Paulo, Brazil; 6 Life and Health Sciences Research Institute (ICVS), School of Health Sciences Universidade do Minho - Campus de Gualtar4710-057 Braga, Portugal; 7 Departamento de Medicina Preventiva, Faculdade de Medicina, Universidade de São Paulo, Sao Paulo, Brazil

## Abstract

**Background:**

The success of HPV vaccination programs will require awareness regarding HPV associated diseases and the benefits of HPV vaccination for the general population. The aim of this study was to assess the level of awareness and knowledge of human papillomavirus (HPV) infection, cervical cancer prevention, vaccines, and factors associated with HPV awareness among young women after birth of the first child.

**Methods:**

This analysis is part of a cross-sectional study carried out at Hospital Maternidade Leonor Mendes de Barros, a large public maternity hospital in Sao Paulo. Primiparous women (15-24 years) who gave birth in that maternity hospital were included. A questionnaire that included questions concerning knowledge of HPV, cervical cancer, and vaccines was applied. To estimate the association of HPV awareness with selected factors, prevalence ratios (PR) were estimated using a generalized linear model (GLM).

**Results:**

Three hundred and one primiparous women were included; 37% of them reported that they "had ever heard about HPV", but only 19% and 7%, respectively, knew that HPV is a sexually transmitted infection (STI) and that it can cause cervical cancer. Seventy-four percent of interviewees mentioned the preventive character of vaccines and all participants affirmed that they would accept HPV vaccination after delivery. In the multivariate analysis, only increasing age (P for trend = 0.021) and previous STI (P < 0.001) were factors independently associated with HPV awareness ("had ever heard about HPV").

**Conclusions:**

This survey indicated that knowledge about the association between HPV and cervical cancer among primiparous young women is low. Therefore, these young low-income primiparous women could benefit greatly from educational interventions to encourage primary and secondary cervical cancer prevention programs.

## Background

Genital infection by oncogenic human papillomavirus (HPV) is a necessary factor in the development of cancer of the cervix [1]. Although the HPV family of viruses includes more than 100 different viral genotypes, HPV 16 and 18 were identified in about 70% of cervical cancer cases [2], while HPV 6 and 11 can cause genital warts [3].

Two prophylactic vaccines to prevent infections by high risk HPV viral genotypes 16 and 18 were available; ideally they should be administered before sexual debut or shortly thereafter to achieve optimal vaccine effectiveness. The US Advisory Committee on Immunization Practices (ACIP) recommended routine HPV vaccination of girls at age 9-12 years and catch-up vaccination for females aged 13-26 years who have not been previously vaccinated or who have not completed the full immunization series [4].

However, providing a vaccine "against cancer" to adolescent girls raises several challenges because many young unmarried girls and women face significant challenges in accessing the health care necessary to meet their sexual and reproductive health needs [5]. For this reason, in some countries school vaccination programs have been established as a strategy to vaccinate girls against HPV. However, pregnant adolescents and girls of lower socio-economic status are more likely to drop out of school, therefore, opportunities for catch-up vaccination are valuable to improve coverage among the at risk young female population.

Then, the success of HPV vaccination programs will require improving awareness regarding HPV, cervical cancer, and the benefits of HPV vaccination for the general population.

Probably, knowledge about HPV has been changing in recent years, given public awareness campaigns around the announcement of the regulatory approval and public announcements of the HPV vaccines.

Thereafter, identifying which groups of women remain unfamiliar with these subjects and would, therefore, benefit from educational messages is crucial, particularly in the target age group for HPV vaccination.

Hence, the aim of this study was to assess the level of awareness and knowledge of HPV infection, cervical cancer prevention, and vaccines, and factors associated with HPV awareness among low-income young women after first delivery in the city of São Paulo, Brazil. The rationale for studying this population is that first delivery health care services could represent a suitable opportunity to offer HPV vaccines for adolescents and young women who have not been previously vaccinated.

## Methods

### Study population

This study is part of a cross-sectional study carried out from June 2006 to February 2007. The study was conducted out at Hospital Maternidade Leonor Mendes de Barros (HMLMB), one of the largest public maternity hospitals in the city of Sao Paulo, Brazil. All primiparous women aged between 15 and 24 years who had been living in the metropolitan area of Sao Paulo for at least six months and gave birth at this hospital after more than 32 weeks of gestation were eligible for the study. The following exclusion criteria, as previously published [6], were applied: non Brazilian, inability or refuse to give informed consent or immunodeficiency (including AIDS/HIV infection checked in medical records).

The study protocol was submitted and approved by the National Ethical Committee (CONEP) (number 188/2006).

Women were recruited to take part in the study during the post-delivery follow-up period in the hospital. Eligible women were contacted by a health professional (a nurse or physician) and asked whether they wanted to know about a study on prevention of cancer of the cervix; those interested had a post-natal visit scheduled within 43 to 60 days after delivery to be enrolled in the study. Women had to attend the post-natal visit and sign the informed consent form to be included in the study.

A total of 509 women were invited to participate: 24 refused, 163 women previously interested in participating did not return to the post-natal visit, and 322 attended the post-natal visit. However, 18 women were not eligible (11 attended the post-natal visit more than 60 days after delivery, one had HIV positive serology, one had more than one delivery, and five were over 24 years old). Three eligible women were excluded by the investigators: one had an acute infectious disease and two aged <18 years lived in a reformatory and had difficulty obtaining a legal representative's signature on the informed consent form within the study period. Therefore, 301 primiparous women were included in the analysis.

### Data collection

During the routine post-natal visit, women were enrolled and interviewed, in an appropriate setting ensuring privacy, by trained interviewers. Using a standardized questionnaire (Additional file 1), information obtained included demographic characteristics, sexual behaviour, reproductive history, contraceptive practice, smoking habits, and awareness and knowledge of HPV, cervical cancer (causes and prevention), vaccines, and HPV vaccine acceptability. Only women who answered questions about awareness of each one of these subjects affirmatively were asked the following open questions, respectively: Could you explain how HPV may be caught and what it may cause? Could you explain what the causes(s) of cervical cancer are? Could you explain how the examination to prevent cervical cancer or "cytological tests" examination is performed? Could you explain what vaccines are? Do you know what vaccines you have been given?

Answers to open questions were grouped in categories according their similarities to be quantified.

### Statistical analysis

Levels of awareness and knowledge of HPV, cervical cancer, and vaccines were expressed as percentages.

To estimate the association of HPV awareness with selected factors, prevalence ratios (PR) and 95% confidence intervals (CI) were calculated, with HPV awareness as the dependent variable and various factors as independent variables. Most independent variables were grouped into two or more categories. For ordered categorical independent variables, tests for linear trend (Chi-square for trend) in the PR were conducted by categorizing the independent variables and entering the continuous scores. Variables selected in univariate analysis at a 0.20 significance level were included in the multivariate analysis. PR and their 95% CI were estimated using a generalized linear model (GLM) with binomial distribution and log link function [7]. Statistical significance was assessed using the likelihood ratio test [8]. A two-sided P value of less than 0.05 was considered to indicate statistical significance. All analyses were performed using STATA version 8.2 [9].

## Results

The mean age of study participants was 19.9 years (median 20.0 years). Over 60% were white and most women (88%) had at least eight years of schooling (the first grade). About two thirds reported incomes (including earnings of all family members) of less than four minimum wages per month (equivalent to US$ 479.20 during the study period).

Awareness of HPV among these young women after first delivery was low: only one third of them reported that they had ever heard of HPV. Among those who had heard of it, less than a quarter knew that HPV can cause cervical cancer; about half knew that HPV is a sexually transmitted infection (STI), and only two participants knew that it can cause genital warts. Only 7% (n = 20) of all participants answered both that HPV is an STI and that it can cause cervical cancer.

When asked about cervical cancer cause(s), very few participants admitted knowing the cause(s) of cervical cancer (8%) and only 6% of women in the sample enumerated HPV as a cervical cancer cause.

Slightly more than a half of the total number of participants referred to knowing about the examination to prevent cervical cancer or cytological tests. However, only 27% answered that in this test some kind of material is collected (a sample of cells from the cervix, cells, secretion, fluid, etc.).

Seventy-four percent of interviewees mentioned the preventive character of vaccines, and only 57% of them answered correctly concerning vaccines that had been given to them (giving the name of at least one vaccine or the name of the disease that the vaccine targeted).

All participants affirmed that they would accept vaccination after delivery if the HPV vaccine were available (Table 1).

**Table 1:** Answers about awareness and knowledge of HPV, cervical cancer (causes and prevention), and vaccines among 301 participants

		Total	%
	**Had ever heard about HPV**	**110**	**37,0**
	**Knowledge about how HPV may be caught and what it may cause**	62	20,6
When asked to explain how HPV may be caught and and what it may cause^1^	HPV is a sexually transmitted disease	58	19,0
	HPV can cause cervical cancer	22	7,3
	HPV can cause genital warts	2	0,7
	Did not know	2	0,7
	**Knowledge about cervical cancer causes**	**23**	**7,6**
When asked to explain what they knew about cervical cancer causes^1^	HPV	19	6,3
	Hereditary disease	1	0,3
	Sexual transmitted disease	1	0,3
	Did not know	2	0,7
	**Knowledge about the examination to prevent cervical cancer or "Pap smear"**	**157**	**52,2**
When asked to explain what "Pap smear" is	Collection of material, secretion, cells, etc.	80	26,6
	Like a gynecological examination^2^	41	13,6
	Repeated the phrase "examination to prevent cervical cancer''	22	7,3
	Other	11	3,7
	Did not know	3	1,0
	**Knowledge about vaccines**	**246**	**81,7**
When asked to explain what they knew about	Prevention of disease	223	74,1
	Treatment	13	4,3
	Other	7	2,3
	Did not know	3	1,0
	**Knowledge about how many vaccines received**	**183**	**60,8**
When asked to explain which vaccines received	Answered at least one vaccine correctly	171	56,8
	Other	7	2,3
	Did not know	5	1,7
	**Acceptance of vaccination against HPV after delivery^3^**	**301**	**100,0**

^1 ^Participants could give more than one answer.

^2 ^Participant seems to know something about it but it is doubtful that she could distinguish it from another gynecological examination.

^3 ^The interviewer first explained what vaccines are and about HPV vaccine.

HPV awareness increased with increasing age (P for trend = 0.003) and with increasing years of schooling (P for trend = 0.039) (Table 2). Table 3 shows the distribution of HPV awareness according to sexual behaviour, reproductive characteristics, and contraception history. Only ever having had a previous STI was significantly associated with HPV awareness (PR = 2.55; 95% CI: 1.93-3.36).

**Table 2:** Prevalence ratios and corresponding 95% confidence intervals (CI) for HPV awareness according to selected socio-demographic characteristics and smoking habits among 301 young primiparous women, Sao Paulo, Brazil, 2006-2007

	Total	HPV awareness	Prevalence ratio	95% CI	P
**Age (years)**					0.003*
**15-18**	90	26.7%	1		
**19-21**	119	35.3%	1.32	[0.87-2.02]	
**22-24**	92	47.8%	1.79	[1.20-2.69]	
					
**Ethnic group**					0.923
**White**	190	37.9%	1		
**Black**	31	32.3%	0.85	[0.50-1.46]	
**Mulatto**	77	35.1%	0.93	[0.65-1.32 ]	
**Indian**	3	33.3%	0.88	[1.76-4.40 ]	
					
**Years of schooling**					0.039*
**≤7**	37	29.7%	1		
**8**	67	28.4%	0.95	[0.51-1.78]	
**9 or 10**	64	35.9%	1.21	[0.67-2.19]	
**≥11**	133	42.9%	1.44	[0.85-2.46]	
					
**Marital status**					0.093
**Living with partner**	245	38.8%	1		
**Single**	56	26.8%	0.69	[0.44-1.10]	
					
**Income^1^**					0.054*
**< 1**	13	38.5%	1		
**1-3**	188	31.9%	0.83	[0.40-1.70]	
**4-6**	80	45.0%	1.17	[0.56-2.43]	
**7-10**	11	27.3%	0.71	[0.22-2.31]	
**> 10**	5	80.0%	2.08	[0.92-4.70]	
					
**Smoking habits**					0.493
**Never**	231	35.5%	1		
**Ever^2^**	70	40.0%	1.13	[0.81-1.58]	

^1 ^In number of minimum wages per month (one minimum wage = R$250.00 or US$119.80; US$1.00 = R$ 2.09, February 2007). Data missing for 4 participants.

^2 ^Included current and former smokers.

*Chi-square for trend

**Table 3:** Prevalence ratios and corresponding 95% confidence intervals (CI) for HPV awareness according to selected sexual behaviours, reproductive characteristics, and history of contraception among 301 young primiparous women, Sao Paulo, Brazil, 2006-2007

	Total	HPV awareness	Prevalence ratio	95% CI	P
**Age at first sexual intercourse**					0.307
**≤15**	132	33.3%	1		
**> 15**	169	39.1%	1.17	[0.86-1.59]	
					
**Number of lifetime sexual partners**					0.238*
**1**	120	37.5%	1		
**2-3**	116	28.5%	0.76	[0.52-1.10]	
**≥4**	65	49.2%	1.31	[0.94-1.84]	
					
**Previous STI^1^**					0.002
**No**	292	34.9%	1		
**Yes**	9	88.9%	2.55	[1.93-3.36]	
					
**Abortion**					0.603
**No**	282	36.2%	1		
**Yes**	19	42.1%	1.16	[0.67-2.02]	
					
**Contraception**					0.398
**No**	74	32.4%	1		
**Yes**	227	37.9%	1.17	[0.81-1.69]	

^1^STI: Sexually Transmitted Infection.

*Chi-square for trend

All selected variables (age, years of schooling, marital status, income and previous STI), were included in the multivariate analysis model. However, when years of schooling and income were included, the model did not converge. Then, the variable income was taken out. In the analysis including the other four variables, years of schooling (P for trend = 0.307) and marital status (P = 0.296) were not significantly associated with HPV awareness. The multivariate analysis revealed that only age (P for trend = 0.021) and previous STI (P < 0.001) were independently associated with HPV awareness. The prevalence ratio of HPV awareness for women aged 19-21 years was 1.32 with a 95% CI of 0.87-2.01, and for women aged 22-24 years it was 1.63 with a 95% CI of 1.07-2.48 (reference group: women 15-18 years old). Women who reported a previous STI were more likely to be aware of HPV (PR = 2.05; 95% CI: 1.46-2.87) compared to women who had never had any STI.

## Discussion

The awareness of HPV among these young primiparous women was low, as only one third of the participants reported having "ever heard about HPV", despite the fact that the present analysis began 16 days after public news and announcements about the first regulatory approval of one of the HPV vaccines, on June 08, 2006 [10], and the fact that participants in the present study had relatively high levels of education within the Brazilian context.

Surveys conducted previously and before the regulatory approval of HPV vaccines also showed a low level of awareness of HPV (30-40%) [11-14]. The results of the present study are in line with other recent studies, carried out after regulatory approval of HPV vaccination, that showed limited levels of awareness of HPV. In the United Kingdom and Italy (where HPV vaccine is free of charge for girls of 12 years of age), only about 24% and 30% of respondents, respectively, reported awareness of HPV [15,16].

In the present study, awareness of HPV was the first question and we did not ask any open questions concerning knowledge of HPV, cervical cancer causes and prevention, or vaccines when the participant was not aware of any of theses subjects, in order to avoid influencing the answers to the open questions or obtaining an answer given correctly by luck, as can occur in studies that use multiple-choice answers.

Only 19% and 7% of the present study participants, respectively, knew that HPV is an STI and that it can cause cervical cancer. Another study, carried out in north-eastern Brazil, assessing young women (16-23 years) showed similar results to the present study: less than 10% of participants acknowledged that HPV might lead to cervical cancer; however, a higher proportion of those women (67%) knew that HPV is sexually transmitted [17]. This difference might be explained by the fact that these women had higher educational levels than women in the present study (61% and 50% respectively had high school education or above). Alternatively, because the authors [17] used five multiple-choice answers per question, a bias cannot be excluded.

We also studied the relationship between HPV awareness and several factors. The multivariate analysis highlights two factors: having had a previous STI and increasing age remained as factors associated with HPV awareness. Accordingly, some authors reported that increasing age (women 14-24 years) [16] and having had a personal, familiar, or friendly history of previous STI or cervical cancer were associated with an increased awareness of HPV and accurate knowledge of the HPV-cervical cancer link [13,14,16].

Half of all participants reported awareness concerning cervical cancer prevention by cytological test, although a smaller number were aware of how it is performed. The query was intended to estimate how many women were aware, and whether they were able to recognize the differences between the ordinal gynecological test and the cytological tests. Based on our data, just 27% of the young primiparous women were able to recognize the differences.

Other studies regarding cytological tests estimated whether the women were aware of their purpose, and hence are not suitable for direct comparisons. They reported a variety of results (10-89%) concerning the interviewees' awareness of the cytological tests' purposes [11,16,17].

In the present study, the awareness regarding vaccines was high, with 74% of the interviewees mentioning their preventative aspect, and 57% of women could identify at least one of the vaccines they had received; that is, they could name the vaccine or the disease which it was intended to prevent.

With regard to the acceptability of the vaccine, despite the inadequate knowledge of HPV and cervical cancer, all participants reported that they would accept vaccination after delivery if the HPV vaccine was available. As other studies have reported, there was a generally favourable attitude toward HPV vaccines; despite the low level of knowledge about the link between HPV and cervical cancer, 91% and 88% of women would agree to receive the vaccine in surveys that found that only 15% [18] and 38% [19], respectively, had heard of HPV.

However, it is important to report that the question about the knowledge of vaccines preceded the question about the acceptability of the HPV vaccine, and for the participants who did not know the answer to the first question, one brief explanation on the prevention of illnesses through vaccines and on the HPV prophylactic vaccine was given by the interviewers in the present study.

In fact, the main factor associated with the acceptance of the HPV prophylactic vaccine in studies carried out prior to its approval was the knowledge of the participants concerning the purpose of vaccines [20,21].

Public acceptance and usage of a prophylactic vaccine are related to the level of knowledge about the disease, which the vaccine will provide protection from [22]. Education about HPV prophylactic vaccines, cervical cancer, and related topics is needed in every country where the vaccine is available.

The main limitation of this study is that 32% of eligible women who were previously interested in participating did not return to the post-natal visit. The likely reason for the relatively large number of women not attending the post-natal visit at our hospital is the availability of post-natal services in public health care centres near their homes, and therefore women may have preferred those for convenience. However, we compared participants and non-respondents using medical hospital records and they did not differ according to several factors: age (P = 0.205), marital status (P = 0.480), smoking habits (P = 0.183), prenatal health care (P = 0.436), and number of prenatal health care visits (P = 0.214). Therefore, this limitation is unlikely to have affected our results significantly.

In Brazil, Quadrivalent and Bivalent HPV vaccines have been approved by regulatory authorities for females aged, respectively, 9 to 26 years and 10 to 25 years but they are not yet included in public vaccination programs [23,24]. Despite the implementation of a national cervical cancer screening program based on cytology in Brazil, about 20,000 cervical cancer cases occur each year [25], indicating a need for revised efforts in education, prevention, and detection.

Although the possibility of knowledge of HPV can be changing in Brazil, probably it has not been changing significantly, because no large educational campaign about HPV for the population has been improved by the government in the last years.

Our findings could add information to knowledge of the Latin American scenario reflecting the views of young women of lower socio-economic status after first delivery. Despite the possibility to provide a large number of information to mothers during the prenatal and postpartum periods, this group of women had low levels of knowledge of HPV and cervical cancer development and prevention. Thus, these women could benefit greatly from educational interventions to encourage participation in primary and secondary cervical cancer prevention programs.

## Conclusions

Young women of low socio-economic status after first delivery had low levels of knowledge of HPV and cervical cancer development and prevention. Thus, opportunities for educational interventions during the prenatal and postpartum periods are valuable to improve knowledge about these subjects.

## List of abbreviations used

(CI): Confidence Intervals; (GLM): Generalized Linear Model; (HPV): Human Papillomavirus; (PR): Prevalence Ratios; (STI): Sexually Transmitted Infection.

## Competing interests

Luisa Lina Villa is a consultant and speaker for the Quadrivalent HPV Vaccine of Merck Sharp & Dohme. José Eluf-Neto has served as a consultant to GlaxoSmithKline in 2006. The others authors have no potential conflicts of interest to disclose.

## Authors' contributions

CHR: participated in study design, coordination during field work, acquisition of data, interpretation of data and helped to draft the manuscript. LLV: conceived of the study, participated in its design and helped to draft the manuscript. SP: conceived of the study, participated in its design and helped to draft the manuscript. MAA: have made substantial contributions to acquisition of data, and helped to draft the manuscript. MCC: have made substantial contributions to acquisition of data, and helped to draft the manuscript. ALA: have made substantial contributions to acquisition of data, interpretation of data, and helped to draft the manuscript. ALF: have made substantial contributions to interpretation of data and helped to draft the manuscript. JEN: participated in the design of the study, performed the statistical analysis and helped to draft the manuscript. All authors read and approved the final manuscript.

## Pre-publication history

The pre-publication history for this paper can be accessed here:

http://www.biomedcentral.com/1472-6874/10/35/prepub

## Supplementary Material

Additional file 1 **QUESTIONNAIRE**. Structured epidemiologic questionnaire with information about demographic characteristics, sexual behavior, reproductive history, contraceptive practice, smoking habits and questions concerning knowledge of HPV, cervical cancer, and vaccines. Click here for file

## References

[B1] Walboomers JMM Jacobs MV Manos MM Bosh FX Kummer JA Shah KV Snijders PJF Peto J Meijer CJLM Muñoz N Human papillomavirus is a necessary cause of invasive cervical cancer worldwide J Pathol 1999 189 12 19 10.1002/(SICI)1096-9896(199909)189:1<12::AID-PATH431>3.0.CO;2-F 10451482

[B2] Muñoz N Bosch FX de Sanjosé S Herrero R Castellsagué X Shah KV Snijders PJ Meijer CJ Epidemiologic classification of human papillomavirus types associated with cervical cancer N Engl J Med 2003 348 518 27 12571259 10.1056/NEJMoa021641

[B3] de Villiers EM Fauquet C Broker TR Bernard HU zur Hausen H Classification of papillomaviruses Virology 2004 324 17 27 10.1016/j.virol.2004.03.033 15183049

[B4] Markowitz LE Dunne EF Saraiya M Lawson HW Chesson H Unger ER Centers for Disease Control and Prevention Quadrivalent human papillomavirus vaccine: recommendations of the Advisory Committee on Immunization Practices (ACIP) MMWR Recomm Rep 2007 56 RR-2 1 24 17380109

[B5] Pollack AE Balkin M Edouard L Cutts F Broutet N WHO/UNFPA Working Group on Sexual and Reproductive Health and HPV Vaccines Ensuring access to HPV vaccines through integrated services: a reproductive health perspective Bull World Health Organ 2007 85 57 63 17242759 10.2471/BLT.06.034397 PMC2636212

[B6] Rama CH Villa LL Pagliusi S Andreoli MA Costa MC Thomann P Alves VAF Longatto-Filho A Eluf-Neto J Opportunity for catch-up HPV vaccination in young women after first delivery J Epidemiol Community Health 2010 64 610 5 10.1136/jech.2008.086439 19692713

[B7] McCullagh P Nelder JA Generalized linear models 1989 2 New York: Chapman and Hall

[B8] Breslow NE Day NE Statistical methods in cancer research The analysis of case-control studies. Publ. no. 32 1980 I 32 Lyon: International Agency for Research on Cancer 7216345

[B9] StataCorp 2003 Stata Statistical Software: Release 8.2 College Station, TX, Stata Corporation

[B10] USA FDA (2006) FDA News Release 8 June 2006 2006 http://www.fda.gov/bbs/topics/NEWS/2006/NEW01385.html (accessed 24 Jan 2007)

[B11] Hanisch R Gustat J Hagensee ME Baena A Salazar JE Castro MV Gaviria AM Sánchez GI Knowledge of Pap screening and human papillomavirus among women attending clinics in Medellín, Colombia Int J Gynecol Cancer 2008 18 1020 6 10.1111/j.1525-1438.2007.01131.x 18021221

[B12] Waller J McCaffery K Forrest S Szarewski A Cadman L Wardle J Awareness of human papillomavirus among women attending a well woman clinic Sex Transm Infect 2003 79 320 2 10.1136/sti.79.4.320 12902585 PMC1744711

[B13] Nøhr B Munk C Tryggvadottir L Sparén P Tran TN Nygård M Skare GB Dasbach E Liaw KL Kjaer SK Awareness of human papillomavirus in a cohort of nearly 70,000 women from four Nordic countries Acta Obstet Gynecol Scand 2008 87 1048 54 18763170 10.1080/00016340802326373

[B14] Tiro JA Meissner HI Kobrin S Chollette V What do women in the U.S. know about human papillomavirus and cervical cancer? Cancer Epidemiol Biomarkers Prev 2007 16 288 94 10.1158/1055-9965.EPI-06-0756 17267388

[B15] Marlow LA Waller J Wardle J Public awareness that HPV is a risk factor for cervical cancer Br J Cancer 2007 97 691 4 10.1038/sj.bjc.6603927 17687335 PMC2360359

[B16] Di Giuseppe G Abbate R Liguori G Albano L Angelillo IF Human papillomavirus and vaccination: knowledge, attitudes, and behavioural intention in adolescents and young women in Italy Br J Cancer 2008 99 225 9 10.1038/sj.bjc.6604454 18628763 PMC2480983

[B17] Moreira ED Jr Oliveira BG Ferraz FM Costa S Costa Filho JO Karic G Knowledge and attitudes about human papillomavirus, Pap smears, and cervical cancer among young women in Brazil: implications for health education and prevention Int J Gynecol Cancer 2006 16 599 603 10.1111/j.1525-1438.2006.00377.x 16681732

[B18] Sauvageau C Duval B Gilca V Lavoie F Ouakki M Human papilloma virus vaccine and cervical cancer screening acceptability among adults in Quebec, Canada BMC Public Health 2007 7 304 10.1186/1471-2458-7-304 17961209 PMC2206027

[B19] Kwan TT Chan KK Yip AM Tam KF Cheung AN Lo SS Lee PW Ngan HY Acceptability of human papillomavirus vaccination among Chinese women: concerns and implications BJOG 2009 116 501 10 10.1111/j.1471-0528.2008.01988.x 19250361

[B20] Lazcano-Ponce E Rivera L Arillo-Santillán E Salmerón J Hernández-Avila M Muñoz N Acceptability of a human papillomavirus (HPV) trial vaccine among mothers of adolescents in Cuernavaca, Mexico Arch Med Res 2001 32 243 7 10.1016/S0188-4409(01)00277-6 11395192

[B21] Davis K Dickman ED Ferris D Dias JK Human papillomavirus vaccine acceptability among parents of 10- to 15-year-old adolescents J Low Genit Tract Dis 2004 8 188 94 10.1097/00128360-200407000-00005 15874862

[B22] Baykal C Al A Uğur MG Cetinkaya N Attar R Arioglu P Knowledge and interest of Turkish women about cervical cancer and HPV vaccine Eur J Gynaecol Oncol 2008 29 76 9 18386470

[B23] Brasil. Ministério da Saúde. Portaria N° 3.124 de 7 de dezembro de 2006 Vacina quadrivalente recombinante contra papilomavírus humanos 2006 Diário Oficial da União; Poder Executivo, de 28 de agosto de ftp://ftp.saude.sp.gov.br/ftpsessp/bibliote/informe_eletronico/2006/iels.dezembro.06/iels236/U_PT-MS-GM-3124_071206.pdf (accessed 20 Dec 2010)

[B24] Brasil. Ministério da Saúde. Resolução -RE No- 474, DE 21 de fevereiro de 2008 Vacina contra HPV oncogênico (16 e 18, recombinante, com advuvante AS04) 2008 Diário Oficial da União; Poder Executivo, de 25 de fevereiro de http://www.anvisa.gov.br/legis/suplemento/250208_suplemento_1.pdf (accessed 02 October 2010)

[B25] Brasil. Instituto Nacional de Câncer [INCA]. Coordenação de Prevenção e Vigilância Estimativa 2008: Incidência de Câncer no Brasil 2008 Rio de Janeiro http://www.inca.gov.br/conteudo_view.asp?id=1793 (accessed 20 Dec 2010)

