# Supplementary material for: Awareness and knowledge of HPV, cervical cancer, and vaccines in young women after first delivery in São Paulo, Brazil - a cross-sectional study
Source: BMC Womens Health. 2010 Dec 22;10:35. doi: 10.1186/1472-6874-10-35 (PMC3022825; doi:10.1186/1472-6874-10-35)
Supplement: Additional file 1 — QUESTIONNAIRE. Structured epidemiologic questionnaire with information about demographic characteristics, sexual behavior, reproductive history, contraceptive practice, smoking habits and questions concerning knowledge of HPV, cervical cancer, and vaccines. [file 1472-6874-10-35-S1.DOC]

**QUESTIONÁRIO**

Nome ______________________________________________________________

Número de identificação no estudo .......................................................................|_|_|_|

Número de registro no Hospital . ..................................................................|_|_|_|_|_|_|

Entrevistadora .............................................................................................................|_|

(1) __________________________________________________________________

(2) __________________________________________________________________

(3) __________________________________________________________________

Data da entrevista ...............................................................................|_|_| |_|_| |_|_|_|_|

dia mês ano

Início da entrevista .........................................................................................|_|_| |_|_|

hora min

Meu nome é _____________________________________ Inicialmente, eu gostaria de agradecer à senhora por participar deste estudo.

Eu irei fazer algumas perguntas que serão registradas neste caderno. Devo dizer que tudo que a senhora responder na entrevista será estritamente confidencial, e as informações colhidas das várias mulheres participantes do estudo serão usadas apenas em relatos científicos, sem nenhuma identificação pessoal.

Os possíveis benefícios deste estudo dependem de que as respostas sejam as mais reais (verdadeiras, sinceras) possíveis. Por favor, pergunte se a senhora não entender o significado de alguma questão. A qualquer momento a senhora poderá recusar-se a continuar ou a responder perguntas específicas. Se houver necessidade de entrar em contato com a senhora, poderia fornecer seu endereço e telefone?

Endereço ____________________________________________________________

Cidade ______________________________________________________________

Telefone ____________________________________________________________

1. Há quanto tempo a senhora mora na região metropolitana de São Paulo?

I_I_I meses ou I_I_I anos

2. Grupo étnico..................................................................................................|_|

(1) Branco (4) Oriental

(2) Negro (5) Indígena

(3) Mulato (6) Outro ______________

3. Qual a sua data de nascimento?..........................................|_|_| |_|_| |_|_|_|_|

dia mês ano

4. Portanto a sua idade (em anos completos) é ........................................... |_|_|

5. A senhora já foi à escola? (1) sim (2) não ............................................... |_|

**[SE RESPOSTA NÃO, VÁ PARA A QUESTÃO 8; SE SIM, CONTINUE]**

6. Qual o nível educacional mais alto que a senhora alcançou? .................|_|

(1) 1º grau incompleto

(2) 1º grau completo

(3) 2º grau incompleto

(4) 2º grau completo

(5) universitário incompleto

(6) universitário completo

(7) outro ___________________

7. Anos de escolaridade (não incluir repetência) ...........................................|_|_|

8. Atualmente a senhora .................................................................................|_|

(1) é casada

(2) tem parceiro regular*

(3) é separada/divorciada

(4) é viúva

(5) é solteira (nunca foi casada nem viveu com parceiro)

****relações sexuais regulares, por pelo menos seis meses, sem contrato legal ou religioso, residindo ou não na mesma casa.***

Agora eu gostaria de fazer algumas perguntas sobre sua vida sexual e reprodutiva.

9. Que idade a senhora tinha quando teve sua primeira relação sexual?.....|_|_|

10. Quantos parceiros sexuais a senhora teve em toda sua vida?................|_|_|

11. Antes desta gestação, a senhora já esteve grávida?..................................|_|

(1) sim (2) não

**[SE RESPOSTA NÃO, VÁ PARA A QUESTÃO 14; SE SIM, CONTINUE]**

12. Quantas gestações a senhora teve?...........................................................|_|

13. Quantas vezes a senhora já abortou?.........................................................|_|

**[PARA AS MULHERES ELEGÍVEIS TODAS AS GESTAÇÕES ANTERIORES DEVEM TER TERMINADO EM ABORTAMENTO]**

14. Que tipo de parto a sra. teve? ....................................................................|_|

(1) parto vaginal

(2) cesariana

15. A senhora fez pré-natal nesta (última) gestação?..........................................................................................................I_I

(1) sim (2) não

**[SE RESPOSTA NÃO, VÁ PARA A QUESTÃO 19; SE SIM, CONTINUE]**

16. Onde a senhora fez o pré-natal? ................................................................I_I

(1) neste hospital

(2) outro serviço/hospital (especifique) _______________________________

17. Quantas consultas de pré-natal a senhora realizou? ..............................I_I_I

18. Aproximadamente quantos meses de gestação a senhora tinha quando fez sua primeira consulta de pré-natal?................................................................. I_I

19. A senhora já usou métodos contraceptivos? .............................................I_I

(1) sim (2) não

**[SE RESPOSTA NÃO, VÁ PARA A QUESTÃO 21; SE SIM, CONTINUE]**

20. Qual método contraceptivo vocês usaram e por quanto tempo?

a) Tabela............................................ |_| por ............ |_|_| meses

b) Coito interrompido........................ |_| por ............ |_|_| meses

c) Preservativo masculino .......................... |_| por ............ |_|_| meses

d) Diafragma...................................... |_| por ............ |_|_| meses

e) Pílula ou injeção ........................... |_| por ............ |_|_| meses

f) Dispositivo intra-uterino (DIU)......... |_| por ............ |_|_| meses

g) Geléia/espermicida......................... |_| por ............ |_|_| meses

h) Outro(s) _______________________ |_| por ............ |_|_| meses

21. A senhora já teve alguma doença sexualmente transmissível?.................|_|

(1) sim (2) não

**[SE RESPOSTA NÃO, VÁ PARA A QUESTÃO 24; SE SIM, CONTINUE]**

22. A senhora foi esclarecida sobre qual (quais) foi (foram) a(s) doença(s)? |_|

(1) sim, foi (foram) _______________________________________________ ______________________________________________________________

(2) não

23. A senhora foi tratada para essa(s) doença(s)? .........................................|_|

(1) sim (2) não

Agora, para as próximas perguntas, por favor tente se lembrar do seu conhecimento anterior sobre o assunto (o que a senhora sabia antes das informações que recebeu ao ser convidada para participar deste estudo).

24. A senhora já tinha ouvido falar do papilomavírus humano (HPV)? ...........|_|

(1) sim (2) não

**[SE RESPOSTA NÃO, VÁ PARA A QUESTÃO 27; SE SIM, CONTINUE]**

25. A senhora sabia como o papilomavírus humano (HPV) pode ser adquirido e o que ele pode causar? ................................................................................|_|

(1) sim (2) não

**[SE RESPOSTA NÃO, VÁ PARA A QUESTÃO 27]**

26**. [Se resposta afirmativa]** Por favor, explique resumidamente como o HPV pode ser adquirido e o que ele pode causar ______________________

______________________________________________________________

27. A senhora sabia qual (quais) é (são) a(s) causa(s) do câncer do colo do útero?...............................................................................................................|_|

(1) sim (2) não

**[SE RESPOSTA NÃO, VÁ PARA A QUESTÃO 29]**

28. **[Se resposta afirmativa]** Por favor, explique resumidamente qual (quais) é (são) a(s) causa(s) do câncer do colo do útero _________________

______________________________________________________________

29. A senhora sabe o que é o exame de “prevenção do câncer do colo uterino” ou exame de Papanicolaou? ...........................................................................|_|

(1) sim (2) não

**[SE RESPOSTA NÃO, A ENTREVISTADORA DEVE EXPLICAR O QUE É O EXAME E IR PARA A QUESTÃO 32]**

30. **[SE RESPOSTA AFIRMATIVA**] Por favor, explique resumidamente o que é o exame de “prevenção do câncer do colo uterino” ou exame de Papanicolaou ___________________________________________________

______________________________________________________________

31. ENTREVISTADORA AVALIA O CONHECIMENTO SOBRE O PAPANICOLAOU ...........................................................................................|_|

1. Sim, ela sabe o que é o teste de Papanicolaou

(2) Ela tem uma idéia, mas é duvidoso que possa distinguir a realização do Papanicolaou de outro procedimento ginecológico

(3) Ela não sabe

**[SE RESPOSTA 2 OU 3, A ENTREVISTADORA DEVE EXPLICAR O QUE É O EXAME]**

32. A senhora já fez exame de “prevenção do câncer do colo uterino” ou exame de Papanicolaou? . ..............................................................................|_|

(1) sim (2) não

**[SE RESPOSTA NÃO, VÁ PARA A QUESTÃO 35, SE SIM CONTINUE]**

33. Quantos exames de “prevenção do câncer do colo uterino” ou exames de Papanicolaou a senhora já fez? ...................................................................I_I_I

34. Há quanto tempo foi seu último exame de “prevenção do câncer do colo uterino” ou exame de Papanicolaou?..........................I_I_I meses ou I_I_I anos

35. A senhora sabe o que são vacinas?.........................................................|_|

(1) sim (2) não

**[SE RESPOSTA NÃO, A ENTREVISTADORA DEVE EXPLICAR O QUE SÃO VACINAS E IR PARA A QUESTÃO 38]**

36. [**SE RESPOSTA AFIRMATIVA**] Por favor, explique resumidamente o que são vacinas ____________________________________________________

______________________________________________________________

37. ENTREVISTADORA AVALIA O CONHECIMENTO SOBRE VACINAS ...|_|

1. Sim, ela sabe o que são vacinas (precisa mencionar o caráter preventivo)

(2) Ela não sabe

**[SE RESPOSTA 2 ENTREVISTADORA DEVE EXPLICAR O QUE SÃO VACINAS]**

38. A senhora sabe quais vacinas recebeu? ...................................................|_|

(1) sim (2) não

**[SE RESPOSTA NÃO, VÁ PARA A QUESTÃO 39]**

**[SE RESPOSTA AFIRMATIVA]** Se sim, por favor, relacione Quais foram as vacinas que a senhora recebeu? ____________________________________

______________________________________________________________

______________________________________________________________

39. Estão sendo desenvolvidas vacinas para prevenir o câncer do colo do útero. Se essas vacinas estivessem disponíveis, a senhora aceitaria vacinar-se após o parto? ..............................................................................................|_|

(1) sim (2) não

40. A senhora é fumante ou já fumou? ...........................................................|_|

(1) sempre foi não fumante

(2) fumante atual (pelo menos um cigarro por dia durante pelo menos um ano)

(3) ex-fumante (fumante anterior que interrompeu o hábito há pelo menos um ano antes da entrevista)*

**** Se interrompeu há menos de um ano considerar como fumante atual***

**[SE RESPOSTA 1, VÁ PARA A QUESTÃO 45; SE 2 OU 3 CONTINUE]**

41. Quantos anos a sra. tinha quando começou a fumar regularmente?... |_|_|

42. Em média, quantos cigarros por dia a senhora fuma ou fumava? .........|_|_|

43. Se a senhora parou de fumar por algum tempo, durante quantos anos a senhora não fumou (no total)? ....................................................................|_|_|

44. (EX-FUMANTE) Quantos anos a sra. tinha quando parou de fumar? .. |_|_|

45. Somando todas as rendas, pensões e salários da família, qual é o ganho mensal familiar (aproximado)?....................................... R$ |_|_|_|_|_|

**[Incluir os ganhos de toda a família]**

45a. [ENTREVISTADORA: SE A MULHER NÃO SOUBER, TENTE ENCAIXÁ-LA EM UMA DAS SEGUINTES CATEGORIAS (GANHO FAMILIAR)] ................................................................................................. |_|

(1) menos de 1 salário mínimo por mês

(2) de 1 a 3 salários mínimos por mês

(3) mais de 3 e até 6 salários mínimos por mês

(4) mais de 6 e até 10 salários mínimos por mês

(5) mais de 10 salários mínimos por mês

46. Quantas pessoas vivem/dependem desse ganho? .......................... |_|_|

Final da entrevista ......................................................................... |_|_| |_|_|

hora min

Muito obrigado (a) por seu tempo e esforço!
